# Retraction Note: Long noncoding RNA lncTCF7, induced by IL-6/STAT3 transactivation, promotes hepatocellular carcinoma aggressiveness through epithelial-mesenchymal transition

**DOI:** 10.1186/s13046-026-03777-5

**Published:** 2026-07-11

**Authors:** Jun Wu, Jun Zhang, Bin Shen, Kai Yin, Jianwei Xu, Wencan Gao, Lihong Zhang

**Affiliations:** Department of Hepatobiliary Surgery, Taixing People’s Hospital, Yangzhou University School of Medicine, 1 Changzheng Road, Taixing, 225400 Jiangsu Province People’s Republic of China

**Retraction Note: J Exp Clin Cancer Res 34, 116 (2015)**

**https://doi.org/10.1186/s13046-015-0229-3**

The Editor-in-Chief has retracted this article.

After publication, concerns were raised regarding some of the images presented in this article, specifically:

- Figure 4 A appears highly similar to Figure S1A in [1];
- Figure 5B appears highly similar to Fig. 3A in [1];
- Figure 5D appears highly similar to Fig. 3C in [1].

The Editor-in-Chief therefore no longer has confidence in the presented data.

Authors have not responded to correspondence regarding this retraction.
